
JOURNAL INFORMATION
==============================
NLM Title Abbreviation: J Child Orthop
Journal ID: J Child Orthop
NLM Journal ID: 101313582

Journal of Children's Orthopaedics

ISSN: 1863-2521
EISSN: 1863-2548
Publisher: SAGE Publications

ARTICLE INFORMATION
==============================
PMCID: PMC3955426
PMID: 24627309
DOI: 10.1007/s11832-014-0574-3
Article ID: 574
Article version: 1
Subjects: Abstracts

EPOS 33rd Annual Meeting

Electronic publication date: 2014 Mar 14
Print publication date: 2014 Apr
Volume: 8
Issue: Suppl 1
First page: 55
Last page: 65
Copyright: © The Author(s) 2014
License: This article is published under license to BioMed Central Ltd. Open Access The abstracts are distributed under the terms of the Creative Commons Attribution License which permits any use, distribution, and reproduction in any medium, provided the original author(s) and the source are credited.
License URL: https://creativecommons.org/licenses/by/4.0/

issue-copyright-statement: © The Author(s) 2014

==============================
J Child Orthop (2014) 8 (Suppl 1):S55–S65
DOI 10.1007/s11832-014-0574-3

ABSTRACTS

### EPOS 33rd Annual Meeting

© The Author(s) 2014. The abstracts are published with open access at Springerlink.com

### PP1

### An anatomical study of the lesser trochanter

**Kathleen Farhang**[1], Claire Shannon[1], Daniel R Cooperman[2], Raymond W Liu[1]

[1]Case Western Reserve University, Cleveland, United States; [2]Yale University School of Medicine, New Haven, United States

**LEVEL 4/Basic science**
Keywords: Lesser trochanter, Femoral anteversion, Anatomic study

**Purpose:** Intramedullary nailing is gaining popularity in treating adolescent femoral shaft fractures, with a reported incidence of rotational malalignment between 20 and 30 % n in adults. The contralateral radiographic lesser trochanter profile can be used as an anatomical landmark to avoid malrotation during intramedullary nailing, but this assumes that the profile and angle of the lesser trochanter are conserved bilaterally.
**Methods:** We studied 1210 paired femora from well-preserved cadaveric skeletons ranging from 22 to 79 years of age at death. Femurs were externally rotated such that the lesser trochanter was parallel to the reference table. The trochanteric angle was measured between the reference table and the bicondylar plane. Lesser trochanter size and shaft diameter were measured with electronic calipers as the largest diameter at the peak and distal base of the lesser trochanter to determine the lesser trochanteric profile. A subset of 196 paired femora were imaged in a neutral position and images used to measure apparent neck shaft angle. Multiple regression analysis was performed to determine bilateral conservation of lesser trochanter profile and angle, and the relationship between anteversion, lesser trochanter angle, and neck shaft angle.
**Results:** Mean age was $56 \pm 10$ years, mean anteversion was $11.3° \pm 12.0°$, mean lesser trochanter angle was $-22.9° \pm 10.9°$, mean neck shaft angle was $125.4° \pm 5.7°$ and mean lesser trochanter profile was $14.2 \pm 4.0$ mm. Multiple regression analysis demonstrated strong correlations between femoral anteversion and lesser trochanter angle (standardized Beta 0.475, $p < 0.0005$).
**Conclusions:** The average femoral lesser trochanter is about 23° posterior to the bicondylar plane, and its profile is about 14 mm. These characteristics are well conserved bilaterally. The angle of the lesser trochanter strongly correlates with both lesser trochanter profile and femoral anteversion.
**Significance:** This study suggests that the angle and profile of the lesser trochanter is conserved bilaterally such that the contralateral femur may be used to estimate proximal rotation to determine malrotation during intramedullary nailing.

### PP2

### Effect of continuous intrathecal baclofen therapy on walking children with cerebral palsy

**Blazej A Pruszczynski**[1], Sees A Julieanne[2], John Henley[3], Chris F Church[3], Keneth J Rogers[2], Freeman Miller[2]

[1]Department of Orthopedics and Pediatric Orthopedics, Medical University of Lod, Lodz, Poland; [2]Department of Orthopedics, Nemours Alfred I. DuPont Hospital for Children, Wilmington, United States; [3]Gait Laboratory, Nemours Alfred I. DuPont Hospital for Children, Wilmington, United States

**LEVEL 4/Cerebral palsy**
Keywords: Intrathecal Baclofen Therapy, Cerebral Palsy, Gait

**Purpose:** Continuous intrathecal infusion of baclofen (CITB) is effective in management of severely involved children with cerebral palsy (CP). However little is known regarding the effect of CITB in ambulatory patients. This study assessed the effects of CITB on function and gait in ambulatory children with CP.
**Methods:** Data were collected retrospectively before and after pump implantation. We compared scores of GMFCS, GMFM, step length, velocity and gait deviation index (GDI). In addition, from the medical chart, the subjective patient opinion regarding the outcome with CITB was summarized. Data were analyzed and summarized using descriptive statistics as well as a two tail *t* test (TT) and paired sample Wilcoxon test (WT).
**Results:** Twenty-seven ambulatory children with cerebral palsy were included. The mean clinical follow-up for all patients was 5 years 3 months (SD: 2 years 3 months, range 1 year–9 years 7 months). The mean follow-up for gait parameters was 2 years 5 months (SD: 1 years 9 months, range 8 months–7 years

6 months) and were available for 19 children. Five patients were quadriplegic, 16 were diplegic, two were hemiplegic and four were dystonic. On the pre-op examination the GMFCS level was as follows: I–2, II–7, III–8, IV–8. For the overall population the GMFM score significantly improved by 4 points from a mean of $14.8 \pm 11.2$ to $18.8 \pm 12.1$ ($p = 0.046$, WT). However, there was no significant change in the group as a whole in gait velocity or GDI. On individual patient assessment, based on medical notes, improvement in gait was noted in 3 of 4 patients with movement disorders. Five of 8 children with pumps implanted before the age of 10 years improved in velocity by greater than 10 cm/s, but only 1 of 10 children with pumps implanted after the age of ten years improved in gait velocity. Three teenage patients discontinued CITB therapy due to weakness and decreased walking endurance (diplegic pattern, GMFCS II and III).

**Conclusions:** CITB treatment did not significantly change the gait parameters in the group considered as a whole set. However younger patients and those with movement disorders demonstrated subjective benefits reported in medical charts. CITB creates weakness, which was not tolerated by teenagers with pure spasticity and was reported by parents. Proper patient selection remains complex and unpredictable.

**Significance:** CITB continues to have potential for positive effects in ambulatory patients if careful patient selection is considered. The flexibility of dose control and the reversibility of treatment make this treatment very adaptive to patient needs.

### PP3

### The contralateral hip joint in unilateral paralytic dislocation. Can we have a conservative attitude? Review of 25 cases

**Joana Ovidio**[1], Miguel Carvalho[1], Nuno Lanca[1], João Lameiras Campagnolo[1], Manuel Cassiano Neves[2]

[1]Hospital Dona Estefânia, Lisboa, Portugal; [2]Hospital Cuf Descobertas, Lisboa, Portugal

**LEVEL 4/Cerebral palsy**
Keywords: Cerebral Palsy, Unilateral Dislocation

**Purpose:** Children with Cerebral Palsy (CP) are at risk of developing dislocation of the hip due to a muscle imbalance around the hip joint. This situation becomes even more apparent in children with severe spasticity and a more marked degree of disability (GMFCS III, IV and V). Some authors believe that surgery to reduce the dislocated hip in the CP must always be associated with surgical procedures on the opposite hip, even if it is reduced, because inevitably there is a risk of their later migration. However, other authors consider that in selected patients, unilateral surgery is enough, not causing harmful effects, in the long term, to the reduced contralateral hip.

**Methods:** We performed a retrospective evaluation of 30 patients, with unilateral hip dislocation, that were submitted to surgery between 1999 and 2013. Two cases were excluded because of lack of radiographs, one by having a contralateral hip disarticulation and two for not having attended the consultations. We characterized groups of patients according to age, gender, aetiology, functional class, and type of surgical procedure performed.

**Results:** We evaluated 25 patients, 13 females and 12 males, aged between 7 and 23 years (average age: 14.5 years) with an average follow up of 4 years (1–14 years) after surgery. All patients presented bilateral spastic CP GMFCS III (7 %), IV (41 %) and V (52 %). Of these children, 10 had associated scoliosis. It was considered in this work that a reduced hip is one that has a migration percentage of less than 30 %. Surgery to the dislocated hip always involved an open reduction with capsulorrhaphy and femoral osteotomy, and in some cases (5) supplemented with acetabular osteotomy. In the hips that were reduced, there has been no surgical procedure, except in four children in which tenotomies of the adductors were performed. At the last visit, 24 children maintained a reduced unoperated hip. One child developed a dislocation of the initially reduced hip, and then was operated three years after the first surgery. The follow up was 0–3 years in 50 % of patients, 4–6 years 38 % 7–9 years at 8 %, and 10 to 13 years at 4 %.

**Conclusions:** Patients with spasticity may present asymmetrical muscle imbalance. Current literature advocates that adduction of a hip can lead to a dislocation, but also we believe that it might, simultaneously, prevent adduction of the opposite hip, preventing its dislocation. Surgery would alter the muscular balance leading a reduced hip but the unoperated hip might dislocate. But some authors think that in some particular cases the unoperated hip will not be dislocated. In this study, of 25 patients, in only one hip was there a gradual hip dislocation, after prior surgery to the hip on the opposite side. More time will be needed in follow up to show the progression of the contralateral hip.

**Significance:** In this analysis, the 25 patients with unilateral dislocation of the hip, operated solely on the affected hip, only one patient had a dislocation of the contralateral hip, that need subsequent surgery. All other patients maintained a migration percentage less than 30 %.

### PP4

### Periacetabular osteotomy(PAO) for the treatment of acetabular dysplasia secondary to Charcot-Marie-Tooth disease(CMT) is associated with a similar clinical improvement but a higher complication rate when compared to Developmental Dysplasia of the Hip (DDH)

**Eduardo Novais**[1], Young-Jo Kim[2], Patrick Carry[3]

[1]Children's Hospital of Colorado, Denver, United States; [2]Boston Children's Hospital, Boston, United States; [3]Children's Hospital of Colorado, Denver, United States

**LEVEL 3/DDH**
Keywords: PAO, CMT, DDH

**Purpose:** Hip dysplasia is estimated to affect 10 % of patients with Charcot-Marie Tooth disease. Hip subluxation, acetabular dysplasia and acetabular anteversion are more severe in CMTHD hips when compared to developmental dysplasia of the hip (DDH). The Bernese periacetabular osteotomy (PAO) is commonly performed for the treatment of symptomatic acetabular dysplasia secondary to DDH. However there is limited evidence to support this procedure in the treatment of CMTHD. The purposes of this study were to compare clinical, functional and radiographic outcomes of PAO among patients affected by hip dysplasia secondary to Charcot-Marie-Tooth disease versus patients affected by classic acetabular dysplasia secondary to DDH.

**Methods:** After IRB approval, a retrospective matched cohort study based on age and gender was used to compare the outcomes of PAO

in patients with symptomatic hip dysplasia secondary to Charcot-Marie-Tooth disease versus classic DDH. Clinical and functional outcomes were assessed by the Harris Hip Scores (HHS). Occurrence of post-operative complications and radiographic correction were compared in the two groups.

**Results:** A total of 89 hips were included in the analysis (31 CMTHD; 58 DDH). There was no difference in gender (p = 0.3551), age at surgery (p = 0.4187), BMI (p = 0.0873), history of a previous hip surgery (p = 0.0738) or duration of follow-up (p = 0.1365) between the two groups. The average duration of follow-up was 5.3 years (range 2 to 16 years). Preoperatively the HHS was found to be significantly [p = 0.0338] lower in the CMTHD group compared to the DDH group. Following surgery, the HHS improved in both groups and there was no significant difference [p = 0.5383] in HHS scores between the two groups at the most recent follow up. The odds of a complication after surgery were significantly higher in the CMTHD group compared with the DDH group (OR: 3.22, 95 % CI: 1.09–9.53; p = 0.0342).

**Conclusions:** At a mean of 5 years, the results of PAO for correction of CMTHD are encouraging and comparable to DDH. Radiographic improvement, improved function and decreased pain after PAO may be expected in CMT patients with symptomatic hip dysplasia undergoing PAO. However, patients with CMTHD are at a significantly higher risk for complications.

**Significance:** Overall, based on evidence of improved radiographic correction and improvements in self-reported hip function and pain, the current study supports the benefits of the Bernese periacetabular osteotomy in subjects with Charcot Marie Tooth Hip Dysplasia.

### PP5

### Development of a bone fixation device for adolescents and adults with small and fragile bones. A preliminary report

**Miguel Angel Galban**, Adolfredo Santana, Carlos Pargas

Centro Clinico Leopoldo Aguerrevere, Caracas, Venezuela

**LEVEL 4/Limb reconstruction**
Keywords: Osteogenesis Imperfecta, Bone Fixation, Endomedular Nail

**Purpose:** Most of the systems for bone fixation are not designed for patients with short stature and small bones with small diameter and length. This condition is common in patients with osteogenesis imperfecta, rickets and bone dysplasia. In children with defects of the bone metabolism, the telescopic nails usually are indicated. The Fassier-Duval Nails have excellent reports, but they work only in patients with open physes, due to the fact that the physes is the structure where the nail is held to the bone.

An endomedulary nail that could fit the sizes of adolescents and adults with small and weak bones did not exist. This deficiency was an incentive for the author to become involved in the design of the GAP NAIL, which has diameters from 4.8 mm and lengths to 320 mm. It is cannulated. It also has the capacity of being locked proximal and distally. It can be used in femur, tibia and humerus.

It is common that these patients have weakness of the lateral cortex of the proximal third of the femur. This is the reason why it was designed to have the possibility to fix the femoral neck and trochanter. This can be achieved with an intramedullary nail linked to a plate via lag and mechanical screws creating a combined Intramedullary/Exomedullary fixation, limiting the risk of stress fractures and improving the implant stability.

**Methods:** We present 5 patients where this system was used in 7 long bones, 6 femurs and one tibia. The ages range from 10 to 48 years. Four patients suffer OI (three patients with type IV and one type III). One patient suffers with Cerebral Palsy (CP) with a severe quadriplegic pattern. Two patients with OI type IV were treated as emergencies due to femoral fractures; one of them had bilateral fractures on the femurs, one patient with OI type III had surgery to the femur and ipsi-lateral tibia l due to severe deformities. One patient aged 48 years with OI type IV had a sub-trochanteric pseudoarthrosis of the femur, and one patient aged 16 years with CP had a severe deformity of the proximal femur, which was the sequel to previous surgeries.

**Results:** The nail‘s smallest diameter was 4.8 mm and the biggest was 8 mm, the length ranged from 200 mm to 320 mm. All nails were locked proximal and distally. In four femurs we use the plate linked to a two lag screws to reinforce the proximal third of the femur. One tibia and one femur were locked proximally and distally with transverse screws. One femur with a fracture needed an extra osteotomy to align the diaphysis. The consolidation was achieved from 8 to 12 weeks, except for the adult patient with a sub-trochanteric pseudoarthrosis which healed in 4 months.

**Conclusions:** In all the cases the objective was reached, which was to stabilize these extremely fragile bones, with diameters below the conventional parameters, without requiring the use of braces postoperatively.

**Significance:** Clinical series

### PP6

### Management of lateral ankle instability in choreo-athetoid patients with cerebral palsy

**Maria Vlachou**, Stefanos Chatzipantelis, Dimitris Dimitriadis

Mitera Children’s Hospital, Athens, Greece

**LEVEL 4/Neuromuscular disorders**
Keywords: Choreoathetosis, Ankle Instability, Cerebral Palsy, Chrisman Snook.

**Purpose:** The study concerns patients with severe uncontrolled choreo-athetoid CP, more prominently expressed with excessive involuntary muscle contractions of the lower limbs, mostly located at the ankle joints forcing them into plantar flexion and inversion. These stressful attacks inevitably lead to the development of lateral ligamentous failure and consequently to instability and chronic painful arthritic changes, with swelling and a varus tilt of the talus in both ankles.

This new technique aims to provide satisfactory results in terms of stability by reconstruction of the lateral ligamentous complex.

**Methods:** Fifteen patients (20 feet) with choreo-athetoid CP and lateral ankle instability, underwent the combination of Chrisman-Snook split brevis graft technique (which is designed to replace both ATFL and CFL and to prevent anterior translation and inversion by restriction of the subtalar motion) with the split tibialis posterior tendon transfer laterally to the Peroneus Brevis (Green technique), in order to reinforce the weakened peroneal action. The mean age of the patients at the time of the operation was 12 years (11–17 years) and the average follow-up was 5 years (2–8 years). All patients were placed in a short leg cast with the ankle at 90°, in a slight eversion and non weight bearing for 6 weeks. Patients were allowed to return to full activities with ankle bracing an average of 4 months after surgery.
**Results:** The outcome of the procedure was satisfactory in 19 feet as the ankle became stable and symptomless, with an unsatisfactory result in 1 foot. On this foot during the surgical exposure, the Peroneus Brevis was found ruptured and markedly frayed along most of its length, therefore making it inappropriate for use as a ligament. As an alternative, half of the Peroneus Longus was used for ligament reconstruction, upon which the transferred split posterior tibial was anchored. Inevitably 3.5 years post-op there was a recurrence of lateral ankle instability and the patient underwent a successful ankle fusion.
**Conclusions:** The results obtained suggest that the application of the combination of these two techniques can be beneficial to a young patient at an early stage of choreo-athetosis with lateral ankle instability, thus avoiding joint fusion.
**Significance:** The combination of the Chrisman–Snook procedure with the split tibialis posterior tendon transfer as a new technique has been successfully employed in 20 consecutive cases of chronic ligamentous instability in children and adolescents with choreo-athetoid CP with very satisfactory overall results and almost no complications.

### PP7

### Changes in the cervical facets orientation during child growth

**Sebastien Pesenti**[1], Tarek Adetchessi[2], Emilie Peltier[1], Karine Chaumoitre[3], Benjamin Blondel[1], Jean-Luc Jouve[1]

[1]Hôpital Timone Enfants, Marseille, France; [2]Hôpital Timone, Marseille, France; [3]Hôpital Nord, Marseille, France

**LEVEL 4/Spine**
Keywords: Cervical Facets Orientation, Spine Growth

**Purpose:** Cervical facets are crucial anatomic elements for both stability and mobility of the cervical spine. During growth, facet orientation compared to the horizontal plane increase progressively. The superposition of these inclined articular joints reinforces spine stability, acting as a mechanical brake during flexion and extension movements. While this anatomical inclination has been frequently observed it has not been clearly reported. The aim of this study was to demonstrate and to quantify the increase of cervical facets orientation during growth.
**Methods:** This study was retrospectively conducted on T1 flash 2D cervical MRI of children aged from 4 months to 18 years old. Eighty children were included. For each vertebra from C3 to C7, facet orientation angle was determined as the angle between the superior articular facet plane and the horizontal, measured on a sagittal slice through the center of the articular process.
**Results:** Extreme values of the orientation angle ranged from 21° to 66°. For each articular process, there was a correlation between facet orientation and age, even if a consequent interpersonal variability was also noticed. In the girls' subgroup, orientation of the facet significantly increased around 11 years old and stopped abruptly at 13 years old. In the boys' subgroup, changes of facet orientation were observed 2 years later and increased after 15 years old, with greater absolute values than for girls. Regardless to age and sex, values of the orientation angle of the C5 facet were lower than the other levels.
**Conclusions:** Results from this study reveal the correlation between age and increase of the fact orientation angle in the cervical spine. According to these normative values, differences were also visible according to gender and vertebral level. The lower values of this angle correspond to the maximum range of cervical flexion–extension.
**Significance:** Level IV

### PP8

### Complications associated with high dose corticosteroid administration in children with spinal trauma

Jason Matthew Cage[1], Jeffrey B Knox[1], Robert Lane Wimberly[2], **Anthony Ian Riccio**[2]

[1]Tripler Army Medical Center, Honolulu, Hawaii, United States; [2]Texas Scottish Rite Hospital for Children, Dallas, Texas, United States

**LEVEL 3/Spine**
Keywords: Spinal Cord Injury, Spine Trauma, Corticosteroid, High Dose Steroids, Complications

**Purpose:** Because of literature to support its use in adults, many trauma centers administer high dose corticosteroids to pediatric patients who have sustained spinal cord injury. Concern has been raised over the medical morbidity of this therapy in adults and early complications following treatment including infection, gastrointestinal bleeding, endocrine abnormalities and wound issues. Little has been published regarding the risks of this treatment for SCI in the pediatric population. The purpose of this study is to determine the incidence of early complications associated with high dose steroid use in pediatric patients.
**Methods:** A trauma registry review was performed to identify all patients treated for spinal injury at a level 1 pediatric trauma center between 2003 and 2011. Medical records were reviewed to identify all patients who received high-dose steroids. A control group consisting of patients with SCI who did not receive steroids was also identified. Demographic data, injury characteristics, and surgical interventions were documented. Complications during the initial hospitalization as well as any complication requiring readmission were identified. Complications were divided into four categories: infectious, gastrointestinal (GI), hyperglycemia/endocrine, and wound problems. Complication rates were compared between the treatment and control groups using a student's t-test and Fischer's exact test.
**Results:** 28 patients (mean age 7.4 years) received high-dose steroid treatment. 11 patients (mean age 8 years) with SCI were identified who did not receive steroid treatment. No statistical difference was detected between the two groups with regard to age, mechanism of injury, rate of surgical intervention, and injury severity via the Revised Trauma Score. Hyperglycemia was the most common complication identified and was present in all patients in both the treatment and control groups. Mean peak glucose was equivalent in both groups (249 mg/dL). The overall infection rate was 65 % in the control group compared to 21 % in the treatment group and this difference was significant.
**Conclusions:** A high rate of acute complications was found in children with spinal trauma, regardless of the administration of high-dose steroids. Hyperglycemia was ubiquitous across both groups suggesting that the administration of steroids may have little effect on worsening pre-existing post-traumatic hyperglycemia in such patients. Though we found a significantly lower rate of infection in children who received high-dose steroids, the reasons behind this are unclear.
**Significance:** High rates of infectious and endocrine complications are present in children with spinal trauma regardless of the administration of high-dose corticosteroids.

### PP9

### Lumbar curve evolution and distal adding-on in Lenke 1 scoliosis

**Walid Lakhal**, Jean Edouard Loret, Joseph Fournier, Charlotte De Bodman, Francois Bergerault, Benoit De Courtivron, Christian Bonnard

Service de Chirurgie Orthopédique et Traumatologique, Hôpital Clocheville, CHRU de Tours, Tours, France

**LEVEL 4/Spine**
Keywords: Adolescent Idiopathic Scoliosis, Adding-On Scoliosis, Lowest Instrumented Vertebra

**Purpose:** Distal adding-on (AO) is a postoperative phenomenon in adolescent idiopathic scoliosis (AIS), which is characterized by a progressive correction loss due to an increase in either vertebral deviation of the lumbar spine or disc angulation below the instrumentation. Distal AO often leads to unsatisfactory clinical outcome and a high risk of reoperation. Selection of the lowest instrumented vertebra (LIV) is recognized as a risk factor of lumbar curve deterioration and occurrence of AO.
Analysis of disc coronal (CROM) and sagittal range of motion (SROM) on dynamic X rays to choose LIV can limit the occurrence of AO.

The objective was to identify the evolution of the lumbar curve after fusion and to study "distal disc ranges of motion strategy" in selection of the LIV.
**Methods:** AIS Lenke 1 treated by posterior fusion with a minimum follow-up of 2 years were included.
The radiographic parameters studied were Cobb angles, flexibility of the curves, apex of the deformity, vertical line erected from the middle of the sacrum (Center Sacral Vertical Line CSVL), stable vertebra, neutral vertebra, distances between CSVL and centroids of LIV and underlying vertebra, and tilt of the upper plate of LIV and underlying vertebra. The SROM and CROM of distal intervertebral disc were measured on dynamic X rays (including right-left side-bending radiographs and full flexion–extension radiographs).
**Results:** Of 185 cases reviewed, 50 AIS Lenke 1 were studied (46 girls; mean age 15 years; thoracic curve of 52°, reducible at 25°; lumbar curve of 30°, reducible of 100 %). Only 3 patients met criteria of adding on, and 2 of them had AO due to intra- or post-operative complications. At last follow up the thoracic curve was 19° with an angular loss of 1.86°. The lumbar curve was 9° with an angular loss of 0.9° at one year, and 1.14° at the last follow up. None of the studied parameters were correlated with the evolution of the lumbar curve.
**Conclusions:** The selection of the LIV remains controversial and optimal positioning is unknown and unpredictable. "Distal disc ranges of motion strategy" in selection of the LIV seems to prevent lumbar curve deterioration and occurrence of AO.
**Significance:** The choice of the LIV based on the analysis of frontal and sagittal disc flexibility seems to prevent AO.

### PP10

### Final fusion after growing rod treatment for early onset scoliosis: is it really final?

**Christina K. Hardesty**[1], Connie Poe-Kochert[1], Claire Shannon[1], Jeff Pawelek[2], George H. Thompson[1], Behrooz Akbarnia[3], David Marks[4], John Emans[5]

[1]Rainbow Babies and Children's Hospital, Cleveland, United States; [2]San Diego Center for Spinal Disorders, San Diego, United States; [3]University of California San Diego, San Diego, United States; [4]Royal Orthopaedic Hospital, Birmingham, United Kingdom; [5]Boston Children's Hospital, Boston, United States

**LEVEL 4/Spine**
Keywords: Early Onset Scoliosis, Fusion, Spine, Pediatric

**Purpose:** "Final fusion" is commonly felt to be the end point for early onset scoliosis (EOS) patients treated with a growing rod (GR). But is it? The purpose of this study was to determine the incidence and cause of reoperation after final fusion for GR patients with EOS.
**Methods:** An IRB approved, multicenter, EOS database was queried to identify GR patients with a minimum of 2 years of follow-up after final fusion. All reoperations were recorded as well their causes. Reoperation was defined as a return to the operating room for any reason related to the EOS.
**Results:** There were 119 potential patients in which 95 (80 %) met the inclusion criteria (34 neuromuscular, 30 syndromic, 23 idiopathic, and 8 congenital patients). The mean age at final fusion was 12 years (range 8 to 18 years) and the average follow-up after final fusion was 4 years (range, 2 to 10 years). Nineteen patients (20 %) had reoperations: 8 syndromic, 6 neuromuscular, 5 idiopathic and no congenital patients. The mean time to first reoperation after final fusion was 2 years (range, 11 days to 7.4 years). Thirty-eight procedures were performed on theses patients: 15 for infection (7 debridement, 4 skin breakdown, 4 instrumentation removal), 8 for failure of instrumentation (5 rod fractures, 3 distal hook or screw pullout), 7 for painful or prominent implants, 2 procedures each for progressive coronal deformity, pseudarthrosis, sagittal decompensation and one procedure each for neurological condition (chiari decompression) and thoracoplasty. The mean number of reoperation for the 19 patients was 2 (range, 1 to 7 reoperations). We did not attempt to determine the current results of these additional procedures.
**Conclusions:** A higher than anticipated percentage of EOS patients with GR treatment required reoperation after final fusion. Long term follow-up after final fusion is, therefore, necessary to determine final results. Further investigation must be done to determine methods for preventing the need for reoperation after final fusion.
**Significance:** Reoperation after final fusion has not been studied before. Parents and patients need to be advised about the possibility of further surgery after final fusion and the importance of long term follow-up.

### PP11

### Spinal deformity in metatropic dysplasia

Mohamed Elkhosousy, **Kenneth Rogers**, Prakash Sitoula, Bober Michael, Mackenzie William

Nemours Alfred I duPont Hospital for Children, Wilmington, United States

**LEVEL 3/Spine**
Keywords: Metatropic Dysplasia, Spine, Diagnosis, Surgery

**Purpose:** Metatropic dysplasia is a rare type of skeletal dysplasia that is associated with a random mutation in the gene encoding transient receptor potential action channel subfamily V member 4 (TRPV4). This mutation results in severe progressive spine and limb deformity associated with significant restrictive pulmonary disease. The aim of this study is to describe the spinal deformity and its management.
**Methods:** Twenty patients with a diagnosis of metatropic dysplasia were identified after a review of the medical records between 1990 and 2013. All patients were seen at our institution. Demographics and treatment progression were collected for all patients. All patients had first and last visit data collected. Pre- and post-operative data was available for all surgical patients.
**Results:** Diagnosis was confirmed after assessment by a medical geneticist and mutation analysis. All patients had thoracic kyphoscoliosis. The average number of spine surgeries was 1.4 (0(5), 1(9), 2(1), 3(4), 5 (1)). Fourteen of 20 patients had an Occiput-C2 fusion and decompression. Seven patients had a posterior spinal fusion and instrumentation for kyphoscoliosis. Complications included incomplete paraplegia in one child and loss of bowel and bladder function in one other. Spinal stenosis is very common. The initial average kyphosis angle at diagnosis of surgical and non-surgical patients was 63.7° and 63° respectively. The surgical group had a higher rate of progression of 1.33°/month compared to 0.15°/month for non-surgical group. For the surgical group, the preoperative mean kyphosis angle pre-op was 89°(70°–98°), and decreased to 70° (52°–86°) at the first post-operative visit, but increased to 84° (65°–112°) at the last visit on record. Three patients are currently being managed with VEPTR growing rod systems after anterior release and halo traction.
**Conclusions:** Children who required surgical management demonstrated a rapid progression of their kyphoscoliosis. Surgical management can be very challenging.
**Significance:** Metatropic Dysplasia patients needs careful monitoring of the progression of spine deformity to determine the appropriate timing for surgical intervention.

### PP12

### Scoliosis in the 22q11.2 deletion syndrome

**Dino Colo**[1], John P. Dormans[2], Elaine H. Zackai[2], Donna M. Mcdonald-Mcginn[2], Denis S. Drummond[2], René M. Castelein[1]

[1]University Medical Center Utrecht (UMCU), Utrecht, Netherlands, The; [2]Children's Hospital of Philadelphia (CHOP), Philadelphia, United States

**LEVEL 2/Spine**
Keywords: Scoliosis, 22q11DS, Spinal Fusion, Complications

**Purpose:** The 22q11.2 deletion syndrome (22q11DS) is a relatively common multisystem disorder, in which the musculoskeletal system may be severely involved. However, there has been little attention in the literature to the orthopaedic disorders in 22q11DS. It seems that scoliosis is the most significant orthopaedic problem. Nevertheless, so far there have only been incidental reports of scoliosis in 22q11DS. The aim of this study was to report the prevalence, characteristics and treatment options of scoliosis in 22q11DS.
**Methods:** A total of 1067 patients from two large 22q11DS-centers (UMCU/CHOP, mean age 11.8 years, (0.8–49), 431 males, 636 females) were included. The UMCU cohort recently started a prospective screening for scoliosis in 22q11DS. A retrospective analysis (partially of prospectively collected data) was performed by reviewing the clinical reports, using questionnaires and assessing available spine X-rays.
**Results:** Scoliosis was found in 18.9 % (F/M 3:2). The curve distribution varied: right thoracic (RT) combined with left lumbar (LL) (27 %), RT alone (17 %), left thoracolumbar (LTL, 9 %), LT (6 %), followed by LT/RTL, RTL and LL. If scored according to the Lenke classification most curves resembled type 1 (32 %), followed by type 3 and 6. Brace treatment was applied in 7 %, however 60 % proceeded to surgery. Surgical correction was required in 25 patients (PSF n = 21, GR n = 5, VEPTR n = 1). 3 patients required 2 procedures because of progression despite surgery. At least 2 more children are planned for surgery. Surgical complications were present in 12 patients (5 hypocalcaemia, 2 excessive bleeding (1 requiring a 2-stage procedure), 3 hardware failure, 1 persistent wound leakage, 1 infection).
**Conclusions:** Scoliosis is very common in 22q11DS (prevalence 20 %). Moreover, the majority of our population have not gone through their growth spurt as yet and are at risk for developing scoliosis. Also, the retrospective analysis of 1 of 2 cohorts may have caused an underestimation of the number of (smaller) curves, as this was also proven by a higher prevalence in the prospectively screened cohort. Surgical intervention was required in 12 %. It appears that brace treatment might be less effective in 22q11DS. It would seem that that a more aggressive fusion is recommended to prevent post-operative decompensation/progression.
**Significance:** Scoliosis is much more common in 22q11DS then previously recognized and can represent a clinically significant problem. We therefore believe that awareness of 22q11DS and its features (among all c-spine anomalies), co-morbidities, syndrome related per-/post-operative complications (e.g. bleeding, hypocalcemia, immune deficiency) is important for every orthopaedic surgeon to know. An extensive preoperative work-up minimizing surgical risks should be standard.
The centres involved in this project are proactively and periodically screening patients for scoliosis collecting their data in a prospective database with the goal of further exploring scoliosis in this syndrome and improving its management.

### PP13

### Serial casting as a delaying tactic in congenital scoliosis

**Gokhan Halil Demirkiran**, Senol Bekmez, Rustem Celilov, Ozgur Dede, Muharrem Yazici

Hacettepe University Orthopedic and Traumatology Department, Ankara, Turkey

**LEVEL 4/Spine**
Keywords: Casting, Congenital Scoliosis

**Purpose:** Several serious concerns exist about early definitive fusion in congenital scoliosis, most serious one being thoracic insufficiency. Because of these concerns delaying tactics such as 'growing rods' and 'VEPTR' techniques have been developed. These modalities subject the patients to multiple surgical interventions and related complications. Therefore, casting techniques have been regaining popularity. The aim of the study is to report the results of extension-derotation-flexion casting techniques in congenital scoliosis.
**Methods:** 12 patients with progressive congenital scoliosis are included (7 girls, 5 boys) in the study. Analyses were done for index casting age, period of follow-up, number of cast applications, index and last Cobb angles, coronal and sagittal balance, requirement for a surgical procedure and cast related complications.
**Results:** All 12 patients had congenital scoliosis with multiple formation and segmentation anomalies. The mean age at index casting was 42.4 months (9–97). Mean pre-casting Cobb angle was 64.6° (22–97). Mean index coronal off-balance was 2.3 cm (0.6–7.4). The mean number of cast applications was 5.5 (3–8). Mean Cobb angle was 47.4° (8–72) after first cast. Mean Cobb angle was 50.7° (4–77) at the last follow-up. Mean coronal off-balance was 1.0 cm (0–2.5) at the last follow-up. No alterations of the sagittal balance were observed. The T1-S1 height was increased from 20.2 cm to 24.1 cm during the treatment period. The requirement of surgery was delayed for an average of 24 months (12–49). Curve progression was observed in three patients. Two patients underwent growing rod surgery. Skin complications were pressure ulcers in three patients. Casts were removed in two patients because of a serious pulmonary infection. These patients were re-casted after recovery. There was no neurologic complications or chest wall deformity in any of the cases.
**Conclusions:** Serial casting is an effective time-buying strategy in order to avoid the morbidity of early definitive fusion or multiple surgical interventions in congenital scoliosis.
**Significance:** This is the first study that evaluates a casting technique as a surgery delaying technique in congenital scoliosis.

### PP14

### The sagittal balance challenge in fusionless surgery: proximal junctional kyphosis (PJK) predicting factors in VEPTR technique

**Norberto M Ventura Gomez**, Anna M Ey Batlle, Augusto Covaro, Imma Vilalta Vidal, David Bongiovanin

Hospital Sant Joan de Déu, Barcelona, Spain

**LEVEL 3/Spine**
Keywords: Sagittal Balance, Fusionless Surgery, Junctional Kyphosis

**Purpose:** To analyse the incidence and risk factors associated with proximal junctional kyphosis (PJK) in patients undergoing instrumented surgery for kyphoscoliosis with fusionless surgery: Vertical Expandable Prosthestic Titanium Rib (VEPTR?)
**Methods:** Clinical and radiographic data on 34 patients (mean age of 6.75 years) from a single center treated with VEPTR with a minimum 2-year follow-up (mean: 4.5 years). Abnormal PJK was defined by a proximal junctional angle greater than 10° and at least 10° greater than the corresponding pre-operative measurement at the beginning of treatment.
**Results:** The incidence of PJK as defined above was seen in 12 patients (35 %). The development of PJK was associated with previous kyphosis T4-T12 > 40° and proximal end vertebral selection from T5 or distally Statistical differences were not founded in non idiopathic patients, prior lumbar lordosis or Cobb angle and failure of distal segment selection. Only 3 of the 12 patients that developed PJK needed additional specific surgery to treat thoracic kyphosis The most common cause of inappropriate end vertebra selection was poor visualization of the upper thoracic vertebra due to prior kyphosis.

Other complications like hardware failure or progressive kyphosis were recorded.
**Conclusions:** Junctional Kyphosis progression can be minimized by the appropriate selection of the upper end vertebra. Improvement of the sagittal balance in previously kyphotic patients could be the most important factor to avoid PJK.
**Significance:** Study of predictive factors of a very difficult to solve complication, in patients with Early Onset Scoliosis treated with VEPTR

### PP15

### Intra- and inter-observer reliability and the role of fracture morphology in classifying femoral shaft fractures in children

**Jeffrey R. Sawyer**[1], Norfleet B. Thompson[1], Derek M. Kelly[1], William C Warner[1], Jeremy K. Rush[2], Alice Moisan[2], David D. Spence[1], James H. Beaty[1]

[1]Campbell Clinic, Memphis, United States; [2]LeBonheur Children's Hospital, Memphis, United States

**LEVEL 3/Trauma—Lower limb**
Keywords: Femur Fracture, Spiral, Non-Accidental Trauma

**Purpose:** Spiral fractures of long bones have long been cited as indications of non-accidental trauma (NAT) in children. However, fracture types are poorly defined in the literature, and intra- and inter-observer variability in defining femoral fracture patterns is rarely mentioned. The purpose of this study is to determine the reliability in classifying femoral shaft fractures in young children using a standard series of radiographs shown to physicians with varied training and experience and to determine if a quantitative approach based objective radiographic measurements could improve definition of these fractures.
**Methods:** On 50 radiographs (AP and lateral) of femoral shaft fractures in children <3 years of age, the fracture ratio—fracture length divided by bone diameter—was determined and radiographs were reviewed by 14 observers who were blinded to the hypothesis of the study, including paediatric orthopaedic surgeons (7), emergency room physicians (5), and musculoskeletal radiologists (2), who classified the fractures as transverse, oblique, or spiral. A second review of the images in a different order was done >10 days after the first. Intra-observer reliability was calculated using the Fleiss kappa statistic and inter-observer reliability was calculated using the Cohen Kappa statistic. Kappa values were interpreted as following: 0.8, nearly complete agreement.
**Results:** Overall, intra-observer agreement was strong (kappa 0.65, range 0.33–0.85). This was highest for radiologists (kappa 0.75) followed by orthopaedic surgeons (kappa 0.71) and ER physicians (kappa 0.54), while inter-observer reliability was moderate (kappa

0.48). Experience level did not correlate with either result. Complete agreement among all observers occurred for only 5 fractures (10 %): 3 transverse and 2 spiral. A fracture ratio <1.4 was predictive of a transverse fracture and a ratio of >3.4, a spiral fracture; ratios between these two values resulted in essentially random classification.
**Conclusions:** The ability to classify reproducibly femoral fractures in young children is highly variable among physicians within the same specialty and between different specialties. These results support the belief that fracture morphology has little predictive value in NAT because of the wide variability in what observers classify as a spiral fracture of the femur.
**Significance:** Caution should be used in the use of descriptive terms such as spiral, oblique, or transverse when classifying femoral fractures, as well as when evaluating children for possible NAT, because of this variability

### PP16

### Transverse femoral shaft fractures are a better predictor of non-accidental trauma than spiral fractures in young children

**Jeffrey R. Sawyer**[1], Ryan Murphy[1], Derek M. Kelly[1], William C. Warner[1], Alice A. Moisan[2], Norfleet Thompson[1], David D. Spence[1], James H. Beaty[1]

[1]University of Tennessee/Campbell Clinic Department of Orthopaedic Surgery, Memphis, United States; [2]Le Bonheur Children's Hospital, Memphis, United States

**LEVEL 3/Trauma—Lower limb**
Keywords: Femur, Fractures, Transverse, Non-Accidental, Trauma, Children

**Purpose:** Spiral fractures of long bones have long been cited as indications of non-accidental trauma (NAT) however recent studies have refuted this. In addition, in most studies there are no strict definitions of a spiral femur fracture. It has been shown recently that a simple morphological method based on plain radiographs, the fracture ratio, can be used to classify these fractures. The purpose of this study is to determine if the fracture ratio can be used to predict whether or not a femoral shaft fracture in a young child is a result of NAT.
**Methods:** We identified 95 consecutive patients over a 7 year period, age <3 years who sustained a closed isolated femoral shaft fracture. Patients with polytrauma, metabolic bone and/or genetic diseases and those with incomplete records/radiographs were excluded. For each patient, their AP and lateral fracture ratios were calculated (fracture length/bone diameter). The lower the fracture ratio, the more transverse the fracture. The presence or absence of a Child Protective Service (CPS) referral, outcome of the referral as well as the Modified Maltreatment Classification Score was recorded. An unpaired T-test was used to compare the groups.
**Results:** Of the 95 patients, 51 patients had a CPS referral (54 %). Of the referred patients there were 13 (25 %) positive and 7 indeterminate (14 %) for NAT. All 20 of these patients had a positive MMCS scoring for NAT. Patients referred to CPS had significantly lower fracture ratios on the AP (1.3 vs 2.6, $p = 0.0037$) and lateral (1.7 vs 2.5, $p = 0.0019$) radiographs. Patients with confirmed NAT had significantly lower fracture ratios on the AP (1.7 vs 2.4, $p = 0.0058$) and lateral (2.6 vs 1.8, p
**Conclusions:** Using a quantitative radiographic measure, the fracture ratio, the more transverse a fracture is on the AP and lateral radiograph the more likely the child is referred to DCS and the more likely the fracture is a result of NAT.
**Significance:** This study has shown that, contrary to numerous citations, the more "spiral" a femoral shaft fracture is, the less likely it is to be as a result of NAT.

### PP17

### Cubitus varus in children: is the lateral close wedge osteotomy complemented by an external fixator a valid method?

**Manuel Cassiano Neves**, Delfin Tavares, Monika Thuesing, Susana Norte Ramos, Francisco Sant'Anna

Hospital CUF Descobertas, Lisbon, Portugal

**LEVEL 4/Trauma—Upper limb**
Keywords: Cubius Varus, Lateral Close Wedge Osteotomy, External Fixator

**Purpose:** Multiple techniques have been proposed to correct a "cubitus varus" deformity, but most of them refer to a high incidence of complications but good functional results. The purpose of this study is to find if the supracondylar lateral closing wedge osteotomy fixed by external fixation + anti-rotatory Kirschner, wires is a valid method to correct the deformity in the 3 planes without complications.
**Methods:** This is a retrospective study analyzing 9 children (6 boys and 3 girls) operated in our institution between January 2012 and June 2013. The average age at the time of surgery was 7.8 years (6–10). Clinically there was an average loss of flexion of 16° and of extension of 21°. The initial deformity measured by the angle of Baumann was 87.3°. All the patients were operated by the same technique: lateral closed wedge osteotomy, medial translation of the distal fragment/correction of rotation + extension deformity and fixation with a "AO external fixator" complemented by Kirschner wires to avoid rotation. The wire was removed at 3 weeks post-op and the External fixator at an average of 4.4 weeks (4–5).
**Results:** There was no vascular complications after surgery and one patient presented a ulnar nerve neuropathy that resolved spontaneously after 6 weeks. There was no infection related to the procedure or the external fixator. Clinically all patients improved their range of motion. The deformity improved to 72.8° and there was no significant translation of the distal fragment or prominence of the lateral condyle. All parents were satisfied with the clinical and cosmetic result.
**Conclusions:** The supracondylar lateral closing wedge osteotomy fixed by external fixation + anti-rotatory Kirschner is a simple operation. The external fixator provides a rigid fixation that allows a full correction of the deformity in the 3 planes with almost no complications.
**Significance:** This simple procedure avoids the complications referred in the literature or the use of more complex techniques

### PP18

### Displaced supracondylar humerus fractures in children: a major trauma centre experience

**Andreas Rehm**, Zeiad Alshameeri, Kuldeep Stohr

Cambridge University Hospitals NHS Trust, Cambridge, United Kingdom

**LEVEL 4/Trauma—Upper limb**
Keywords: Supracondylar Humerus Fracture

**Purpose:** To assess management and outcome of displaced supracondylar humerus fractures in children in a mixed adult and paediatric major trauma centre setting.
**Methods:** Radiographs and records of all children who were treated with a displaced supracondylar humerus fracture at our hospital between 2005 and 2012 were reviewed. The radiographs were assessed for varus/valgus malalignment, the position of the anterior humeral line in relation to the capitellum and malrotation. Information on surgery and complications were collected. A questionnaire was sent to all parents asking them to document elbow movements and alignment on provided drawings and to comment on function and symptoms.
**Results:** 147 patients with 148 displaced supracondylar fractures were treated. The mean age at injury was 79 months with a mean follow up of 54 months. 17 open and 128 closed reductions were performed. In 3 a plaster was applied without reduction. Seven different wire configurations were used. 58 different surgeons were involved. 31 procedures were performed without assistant and 53 by a senior trainee without a consultant. Ten patients required 11 additional procedures including 2 ulnar nerve repairs, 2 valgus osteotomies and 7 re-manipulations. Only 1 pin site infection and 1 over-granulation were recorded.
In 50 fractures there was either incomplete reduction or some loss of reduction. Radiographs showed varus malalignment in 10, the anterior humeral line missing the capitellum in 32 and a combination of both in 8 fractures but the parents of 33 of these patients reported normal alignment and movement. 92 questionnaires were completed. In 47 patients parents reported abnormal alignment and/or movement despite acceptable radiological criteria in 26 of these. 7 parents were concerned about symptoms or alignment including one ulnar nerve injury patient who still had weakness 5 years after surgery.

**Conclusions:** Re-displacement, incomplete reductions, secondary surgery rate and asymmetric movement and/or alignment at follow-up in supracondylar humerus fractures are high in our series which was independent of the seniority of the surgeon. There was no correlation between post-operative radiological alignment and parental assessment of appearance and movement. Parent/patient satisfaction was high.
**Significance:** This study supports that displaced supracondylar humerus fractures in children can be managed in a non paediatric orthopaedic setting by fully trained orthopaedic surgeons and senior trainees. The criteria used to define an acceptable reduction do not predict normal alignment and normal range of movement as assessed by our parents. Our study indicates that the criteria used as outcome measures in the literature might have to be redefined.

### PP19

### Reconstruction using induced membrane technique after segmental bone resection of primitive malignant bone tumours in children

**Camille Thevenin-Lemoine**, Pierre Mary, Manon Bachy, Franck Fitoussi, Raphael Vialle

Hôpital Trousseau, Paris, France, Metropolitan

**LEVEL 4/Tumors and metabolic disorders**
Keywords: Bone Tumor, Induced Membrane

**Purpose:** The induced membrane technique has proven its interest in the reconstruction of traumatic bone loss. Resection of primary malignant bone tumour in children often leads to challenging lack of substance. We report our experience using this technique after resection of primary bone tumors in children.
**Methods:** We reviewed all the patients who underwent segmental resection for primary bone tumor with reconstruction using the technique of induced membrane with a minimum follow-up of 2 years
**Results:** Thirteen cases have been collected. There were 5 cases of osteosarcoma, 7 Ewing sarcoma, and 1 undifferentiated sarcoma. The mean age at the time of resection was 10 years old (range 9 months to 17 years). The mean length of resection was 117 mm (60 to 220 mm). In addition to the cement spacer, osteosynthesis was most often carried out by locking screw plate (8 cases). Partial weight bearing was authorized immediately in all cases and full weight bearing after two months on average. Adjuvant chemotherapy was restarted in an average of 14 days after resection. The bone graft was performed an average of 2 months after the end of adjuvant chemotherapy, or an average of 8 months after the resection surgery. It was in all cases of posterior iliac cancellous autograft mixed with granules of tricalcium phosphate and associated with a tibial strut in 4 cases.

Two scar necrosis occurred early after the first surgery. There were 4 non-unions. In three out of four cases, consolidation was obtained after a second autologous bone graft. One tibial varus deviation was corrected by osteotomy with external fixator in the graft area, allowing the simultaneous correction of length discrepancy. No case of massive osteolysis of the graft or deep infection was observed.

Early loading of the graft, facilitated by the use of locking screw plates, seems to limit the risk of non-union and avoided the occurrence of massive osteolysis. The initial reconstruction procedure with cement shorten the surgery, as it does not require microsurgical skills, and allows early resumption of chemotherapy with limited risk of infection. Reconstruction with a “living” bone is a guarantee of its long term sustainability.
**Conclusions:** Despite the small number of patients and a large variability of bone defects treated, the induced membrane technique appears to be reliable for reconstruction after resection of a primary bone tumor in children.
**Significance:** Level 3

### PP20

### Radial head resection and hemi-interposition arthroplasty or corrective osteotomy in Masada type 2 deformity in patients with multiple hereditary exostoses. Evaluation of a new operative technique

**Konrad Mader**[1], Arnard Van Der Zwan[2], John Ham[2]

[1]Sentralsykehuset, FøRde, Norway; [2]OLVG, Amsterdam, Netherlands, The

**LEVEL 4/Tumors and metabolic disorders**
Keywords: Multiple hereditary exostoses, Radial head deformity, Correction osteotomy, Interposition arthroplasty, External fixation

**Purpose:** Forearm osteochondromas and/or deformities are found in a vast majority of patients diagnosed with multiple hereditary exostoses (MHE). In Masada type II in addition to osteochondroma formation the radial head is chronically dislocated/subluxated from the elbow joint with massive compromise to both elbow stability and forearm movement. In a prospective protocol the value of corrective osteotomy of the radial head and neck and a newly developed surgical technique with radial head resection, capitulum hemi-interposition arthroplasty, radial ligament augmentation and hinged external fixation were evaluated.
**Methods:** Five girls and eight boys were included into the study with a mean age of 13 years (range, 8 to 16 years of age). In 2 cases, after forearm leveling with correction of the ulnar length and radial deformity in an earlier stage, a 3-dimensional correction of the multiplanar deformity of the radial head and neck was performed in order to correct the chronic proximal head dislocation. In 10 cases (one after failed corrective osteotomy with secondary dislocation of the radial head) a new operative procedure, namely a radial head resection combined with a hemi-interposition arthroplasty using the internal layer of the widened capsule with parts of the deep layer of the common extensor origin over the capitulum, ligament reconstruction/augmentation of the radio—ulnar collateral ligament complex and hinged external fixation was performed by the senior authors. In 2 other cases hinged fixation was not necessary due to satisfactory radial stability of the elbow after radial ligament stabilization. External fixation was removed after 6 weeks, function, pain, patient satisfaction, patient-related outcome and radiologic appearance of the elbow and the wrist joint was recorded at a minimum-follow-up at 12 months (range, 12 to 46 months).
**Results:** There were no procedure complications, especially no injury to the deep branch of the radial nerve or fixator-related problems. All three corrective osteotomy patients showed major deficits in forearm rotation, pain during motion and radial head dislocation at latest follow-up. One patient opted for revision to radial head resection and interposition at 1 year—postoperative. The two other patients are staged to the radial head resection procedure in the near future. The ten patients in the radial head resection treatment group improved substantially in forearm rotation, patient satisfaction, pain (VAS) and patient related outcome measure. Of importance, no Essex-Lopresti phenomen was detected during follow-up. In two patients there was minor pain after strenuous exercises in the elbow and proximal forearm recorded. All patients were satisfied with the procedure. In one patient a regrown osteochondroma in the proximal radio—ulnar joint led to a revision procedure and osteochondroma removal at a later stage. All elbows were stable.
**Conclusions:** Patients with Masada type II deformities in the elbow do not gain profit from the attempt to correct the radial head length and axis during intermediate follow-up. The use of radial head resection, stabilization of the radial ligament complex, interposition arthroplasty with hinged external fixation shows promising patient related and radiologic outcome. It is a clear option for chronic radial head dislocation in the patient group after leveling of the forearm length. It is a demanding procedure and should be performed in dedicated centers.
**Significance:** It is a clear option for chronic radial head dislocation in the patient group after leveling of the forearm length. It is a demanding procedure and should be performed in dedicated centers.

### PP21

### Congenital volkmann's ischemic contracture: a rare and challenging condition

**Marisa Cabrera GonzáLez**, Laura Montserrat Pérez López

Sant Joan de Déu Children's Hospital, Barcelona, Spain

**LEVEL 4/Upper extremity**
Keywords: Volkmann, Ischemic Contracture

**Purpose:** Congenital Volkmann's ischemic contracture is a very uncommon situation. Only around fifty cases have been described in literature.
In order to minimize the devastating consequences of this condition, a therapeutic approach may be systematized.

**Methods:** Five patients have been evaluated from 1998 to 2013. In all cases, unilateral hand and forearm were affected. The management and evolution of these cases is described, and a review of literature is made.

The pathological findings during pregnancy were: one gestational diabetes with regular insulin treatment, one pre-eclampsia and three unspecific oligoamnios.

The association of skin lesions (necrotic bedsore with a central pallor, ulcers, bullae), diffuse oedema, contractures and neuromuscular affectation (flaccid paralysis) in a newborn's upper limb leads to the diagnosis of congenital Volkmann's ischemic contracture. This presentation should not be confused with aplasia cutis congenital, septic necrotizing fasciitis, amniotic band syndrome, epidermiolysis bullosa, neonatal gangrene, congenital varicella or vascular affectation.

Radiological findings such as demineralization, metaphyseal rarefaction, epiphysis affectation or even more extensive diaphysis bone affectation may be found.
**Results:** An urgent approach was proposed, based on the common surgical procedures for any Volkmann's contracture: fasciotomy, wide skin and deep tissue's debridement, neurolysis, scar release and skin graft. In two cases, we included the use of a regional anaesthetic axillary block with ropivacaine. The regional anaesthetic caused a sympathetic block, which generated a vasodilatating response.
Later, we recommended hand therapy and splinting. Also some deferred surgical procedures were needed, such as contracture releases, tendon transfers, angular deformities bone corrections and neural releases. In one patient, a digit amputation was mandatory.

We evaluated wrist, metacarpo-phalangeal and inter-phalangeal mobility, as well as the thumb function with good functional results in three patients. One patient presented a deep mobility affectation. Another patient was lost during the follow-up.
**Conclusions:** There is a clear connection between some extrinsic intra-uterine and intrinsic fetal factors during the last trimester of pregnancy and an increased intra-compartmental pressure in these affected upper limbs.
These patients require an urgent treatment, which may minimize late consequences such as disrupted upper limb functionality and, in some cases, even amputations.

The use of a regional anesthetic block, which causes a sympathetic block and with that, a vasodilatation, seems to improve substantially the final functional results.
**Significance:** Efforts among the community of pediatric hand surgeons may be joined, in order to share knowledge and to provide new ideas. The origin and the possible treatments for this devastating condition need more study and detailed analysis.

### PP22

### What role do plain radiographs have in assessing the skeletally immature acromio-clavicular joint?

**Moon Seok Park**[1], Kyoung Min Lee[1], Soon-Sun Kwon[1], Seung Yeol Lee[1], In Hyeok Lee[1], Sang Hyeong Lee[2], Dae Gyu Kwon[3], Chin Youb Chung[1]

[1]Seoul National University Bundang Hospital, Seong Nam, Korea; [2]Dongguk University Ilsan Hospital, Go Yang, Korea; [3]In-Ha University Hospital, In Cheon, Korea

**LEVEL 4/Upper extremity**
Keywords: Acromioclavicular Joint, Coracoid Process, Acromion, Incomplete Ossification

**Purpose:** Because of incomplete ossification of the coracoid process and acromion, acromio-clavicular joint configuration in the skeletally immature patient differs from adults. This study was performed to establish normal anatomy on conventional radiograph of the shoulder in skeletally immature bone.
**Methods:** This study was based on a total of 485 subjects who underwent conventional radiographs of bilateral shoulder antero-posterior view at age of 8 to 18 year old. The progression of ossification of acromion and coracoid process divided into 3 groups (none, partial, and complete) on radiographs was evaluated. The vertical and shortest coraco-clavicular interval, coraco-clavicular clavicle width ratio, acromio-clavicular joint offset, and difference of the coraco-clavicular interval of both sides were measured. A reliability test was conducted prior to main measurement. The relationship of measurements with gender, age and stage of ossification was evaluated.
**Results:** Classification of ossification of the acromion and the coracoid process, vertical and shortest coraco-clavicular interval showed excellent reliability (intra-class correlation coefficient, 0.868, 0.886, 0.918, and 0.934). The acromio-clavicular joint offset showed low reliability (intra-class correlation coefficient, 0.543). Ossification of the acromion tends to appear earlier and to fuse to scapula later than the coracoid process. The vertical coraco-clavicular interval was less than 11 mm in 90 % of total subjects in males, and 10 mm in 90 % in females. The vertical coraco-clavicular interval was not affected by partial ossification of the coracoid process. The difference of vertical coracoclavicular interval of both sides was less than 50 % in 93.4 % of total subjects.
**Conclusions:** The vertical coraco-clavicular interval was initially applicable to assess acromio-clavicular joint dislocation in skeletally immature patients in terms of reliability and influence of ossification. Comparison of both sides of the acromio-clavicular joint could help to inform physicians in predicting the need for further evaluations.
**Significance:** This study demonstrated that vertical coraco-clavicular interval was useful indication of acromio-clavicular joint dislocation in skeletally immature patients.

### PP23

### Congenital pseudarthrosis of the clavicle: the "Istituto Ortopedico Rizzoli" experience

Giovanni Luigi Di Gennaro, **Alessandro Martinelli**, Mattia Cravino, Diego Antonioli, Onofrio Donzelli

Istituto Ortopedico Rizzoli, Bologna, Italy

**LEVEL 4/Upper extremity**
Keywords: Congenital Pseudoarthrosis, Clavicle

**Purpose:** Congenital pseudoarthrosis of the clavicle is a rare condition. The aetiology remains unknown and treatment is still controversial. In order to add some insight into the treatment method of this pathology we performed a retrospective search in the archive of Rizzoli Orthopaedic Institute in Bologna.
**Methods:** We found 22 children (10 M; 12 F) treated surgically between 1960 and 2012. We have evaluated the clinical and radiographic characteristics of every case. The surgical technique was analyzed for every case.
The surgical technique was similar for all patients, consisting in removal of the pseudarthrotic tissue, autologous iliac bone graft and K wire fixation. In one case a plate was used instead of the K wire. In two cases allograft bone was used instead of autologous iliac bone graft. All patients were treated postoperatively with a shoulder spica cast.
**Results:** Follow-up ranged from 6 months to 19 years. All patients were pain free, range of motion was complete, and no other subjective anomalies were found. None of our patients reported vascular or neurological symptoms. X-rays showed a typical pattern consisting in a pseudarthrotic lesion of the mid portion of the clavicle. The distance between the segments ranged from 2 to 14 mm with a mean gap of 7 mm.
Bone healing was achieved in 16 cases between 8 and 16 weeks after the operation. Post-surgical immobilization with a shoulder spica cast was maintained for 8 to 16 weeks.
A post surgical infection was observed in the patient treated with plate. A second surgical procedure for wound debridement and hardware removal was performed. In both the two cases treated with fibular allograft bone we observed a recurrence of the pseudarthrosis.
**Conclusions:** According to our experience we believe that congenital pseudarthrosis of the clavicle needs an early surgical treatment between four to ten years of age, in particular in patients with great deformity.
Surgical treatment should focus on an accurate removal of the pseudarthrotic tissue; special care should be taken in shaping and positioning the graft that, according to our experience, should be autologous bone harvested from the iliac crest. Furthermore the graft should be ideally shaped as a barrel made from cortical scales, filled with cancellous bone. The construct should be fixed with a K wire in order to minimize interference with natural bone healing process. Alograft bone alone is not enough to achieve bone healing.
The use of a cast for at least 8 weeks after surgery is the essential requirement for a successful treatment.
**Significance:** This paper is a retrospective review of twenty-two cases of congenital pseudarthrosis of the clavicle treated in our institution. This case series is one of the largest present in literature.

**Open Access** The abstracts are distributed under the terms of the Creative Commons Attribution License which permits any use, distribution, and reproduction in any medium, provided the original author(s) and the source are credited.
